# P-Rex1 Signaling Hub in Lower Grade Glioma Patients, Found by *In Silico* Data Mining, Correlates With Reduced Survival and Augmented Immune Tumor Microenvironment

**DOI:** 10.3389/fonc.2022.922025

**Published:** 2022-07-07

**Authors:** Yarely Mabell Beltrán-Navarro, Guadalupe Reyes-Cruz, José Vázquez-Prado

**Affiliations:** ^1^Department of Pharmacology, Cinvestav-IPN, Mexico City, Mexico; ^2^Department of Cell Biology, Cinvestav-IPN, Mexico City, Mexico

**Keywords:** P-Rex1, LGG, tumor microenvironment, cancer biomarker, prognostic signaling signature, RhoGEF, signaling hub

## Abstract

Systematic analysis of tumor transcriptomes, combined with deep genome sequencing and detailed clinical assessment of hundreds of patients, constitutes a powerful strategy aimed to identify potential biomarkers and therapeutic targets to guide personalized treatments. Oncogenic signaling cascades are integrated by multidomain effector proteins such as P-Rex1, a guanine nucleotide exchange factor for the Rac GTPase (RacGEF), known to promote metastatic dissemination of cancer cells. We hypothesized that patients with high P-Rex1 expression and reduced survival might be characterized by a particular set of signaling proteins co-expressed with this effector of cell migration as a central component of a putative signaling hub indicative of poor prognosis. High P-Rex1 expression correlated with reduced survival of TCGA Lower Grade Glioma (LGG) patients. Thus, guided by PREX1 expression, we searched for signaling partners of this RacGEF by applying a systematic unbiased in silico data mining strategy. We identified 30 putative signaling partners that also correlated with reduced patient survival. These included GPCRs such as *CXCR3*, *GPR82*, *FZD6*, as well as *MAP3K1*, *MAP2K3*, *NEK8*, *DYRK3* and *RPS6KA3* kinases, and *PTPN2* and *PTPN22* phosphatases, among other transcripts of signaling proteins and phospho-substrates. This *PREX1* signaling hub signature correlated with increased risk of shorter survival of LGG patients from independent datasets and coincided with immune and endothelial transcriptomic signatures, indicating that myeloid infiltration and tumor angiogenesis might contribute to worsen brain tumor pathology. In conclusion, P-Rex1 and its putative signaling partners in LGG are indicative of a signaling landscape of the tumor microenvironment that correlates with poor prognosis and might guide the characterization of signaling targets leading the eventual development of immunotherapeutic strategies.

## Introduction

RhoGEFs are signaling proteins that respond to spatiotemporal cues that drive cell migration (1, 2). Composed by multiple functional domains, they are hierarchically positioned to integrate pro-metastatic protein complexes that might worsen the prognosis of different types of cancer (3, 4). P-Rex1, in particular, is a multidomain effector of phosphoinositides and heterotrimeric G proteins, acting *via* Gβγ, involved in cell migration and metastatic dissemination of cancer cells (5–9). It activates Rac, a small GTPase of the Rho family that promotes cell protrusion by leading the assembly of actin polymers into lamellipodia at the cell front (6–9). As a metastatic RhoGEF, P-Rex1 contributes to the dissemination of breast, melanoma and prostate tumors to bones, lungs and lymph nodes, respectively (5–9). In experimental murine gliomas caused by autocrine PDGF signaling, *PREX1* was found upregulated as a putative promoter of cell transformation (10, 11). In primary cells from glioblastoma patients, highly expressed P-Rex1 was found playing an important role in cell invasion (12). This RhoGEF was identified as an upregulated target of *ZEB1*, a transcription factor known to promote epithelial-mesenchymal transition (13). *PREX1* and *ZEB1* are co-expressed in glioblastoma patients in which high P-Rex1 correlates with reduced survival (13). P-Rex1 is also highly expressed in lower grade glioma patients (LGG); however, it is currently unknown whether this is indicative of bad prognosis, as in the case of glioblastoma and other cancers (13).

Classification of brain tumors, traditionally carried out using histological methods, has been further refined according to their genetic alterations (14, 15). They include LGG, a slow growing non-invasive cancer, characterized as grade II-III and highly invasive grade IV glioblastoma, which can arise from LGG (16–18). In the case of diffuse gliomas, the histological observation of transformed glial cells initially defined the subtype as astrocytoma, oligodendroglioma or oligoastrocytoma, which in the Cancer Genome Atlas (TCGA) correspond to 37.7%, 36.8% and 25.3%, respectively (19, 20). More recently, molecular diagnostics have reclassified oligoastrocytomas into astrocytic or oligodendroglial tumors (21). Characterization of genomic alterations has served to establish three main groups of glial cancers according to isocitrate dehydrogenase *(IDH1/2*) mutations and chromosomic 1p/19q codeletion status: mutant *IDH*-non-codel, mutant *IDH*-codel and wild type *IDH* (22–25). Additional classification is based on loss of nuclear ATRX chromatin remodeler (*ATRX)*, telomerase reverse transcriptase (*TERT)* expression and mutations within its promoter, and several DNA methylation subgroups (G-CIMP, Glioma CpG island methylator phenotype) (23, 26–28). Different mutated genes also contribute to the classification, like *CIC*, *FUBP1* and *TP53* (23, 27), *H3F3A* (29), *EGFR* and *PTEN* (30). *MGMT* promoter methylation status is considered a prognostic marker that helps to decide treatment (31). Further characterization of LGG biomarkers that might define clinical pand guide patient treatment can be achieved by assessing the transcriptomic landscape of individual tumors (32–37). In this regard, considering that signaling circuits might be postulated as oncogenic signatures whose characterization as integrated networks, composed by a repertoire of receptors, transducers, and effectors, might be indicative of vulnerabilities and prognosis, here we pursued a systematic data-mining strategy aimed to characterize the repertoire of P-Rex1-linked signaling proteins that correlate with bad prognosis of LGG patients.

## Materials and Methods

### TCGA Datasets, Processing, Analysis and Validation

TCGA datasets: LGG (low grade glioma, 510 patients), LAML (acute myeloid leukemia, 150 patients), KIRC (kidney renal clear cell carcinoma, 522 patients), and LUAD (lung adenocarcinoma, 492 patients), including gene expression data (mRNA expression RSEM, batch normalized from Illumina HiSeq_RNASeqV2), the fraction of altered genome, and clinical data, were obtained from the cBioPortal database for Cancer Genomics (https://cbioportal.org/). *PREX1* expression/survival curves in 21 TCGA datasets were analyzed in the http://www.oncolnc.org/ platform. Comparative survival curves of LGG patients selected by low and high *PREX1* expression (50% each group), or that of signaling transcripts identified by their high coexpression with *PREX1* in the group of patients highly expressing this RacGEF, were analyzed with the Logrank test in the OncoLnc platform and graphs were prepared with GraphPad Prism 6.01.

#### Genetic Dependencies and Small Molecule Sensitivities in Cancer Cell Lines

To identify cell lines in which *PREX1* and its potential signaling partners were vital, *PREX1* essentiality profile was first analyzed in the Cancer Dependency Map platform: https://depmap.org/portal/gene/PREX1?tab=dependency (perturbation effects tab). Cells corresponding to the cancer types in which *PREX1* expression correlated with patient survival, with T-statistic values of -0.5 or more negative, were selected for further analysis. From the astrocytoma context, in which *PREX1* expression was linked to cell viability, the list of essential genes and drug sensitivities was downloaded to identify those coding for signaling proteins (https://depmap.org/portal/context/astrocytoma). Given that only a small set of cancer cells depended on P-Rex1, it was expected that signaling partners of this RacGEF were identifiable as coessential genes. These potential P-Rex1 signaling partners were tagged by merging the list of essential genes with a list of human signaling proteins obtained from the SMART (http://smart.embl-heidelberg.de/) and UniProt (https://www.uniprot.org/) platforms.

The DEPMAP platform contains a genome-scale catalog of genetic vulnerabilities and sensitivity to small molecules based on systematic synthetic lethality screenings, aiming to identify essential genes critical to cancer cell survival and proliferation (38, 39). As such, it is as an unbiased source of potential targets of precision medicine, as it includes synthetic lethality data of thousands of genes, including those coding for intracellular signaling proteins such as *PREX1* and its signaling partners, which might be coessential in specific survival and proliferative pathways. In general terms, selective gene essentiality in particular cancer cells points to potential drug targets with fewer collateral effects, since these pathways are not linked to fitness in most cells (putatively including normal cells). Available genome-wide RNAi and CRISPR loss-of-function screens and drug sensitivity screens were included in our analysis.

#### Transcripts of Signaling Proteins Coexpressed With PREX1

Genes highly coexpressed with *PREX1* in tumors were identified from TCGA datasets based on the list of Spearman’s correlation coefficient values ordered from the highest positive to the highest negative. Specifically, from the whole group of TCGA LGG patients, the list of genes coexpressed with *PREX1* was obtained from the cBioportal platform: (https://www.cbioportal.org/results/PREX1coexpression_list=lgg_tcga). Two groups of TCGA LGG patients were independently analyzed as the high and low *PREX1* expression groups, aiming to identify genes differentially coexpressed with *PREX1* in the high expression group. Patient identification and order, based on *PREX1* expression, was established in the http://www.oncolnc.org/ platform. Data of coexpressed genes were downloaded and organized in quartiles from highest positive to negative Spearman values. Those in the top quartile, with the highest positive values, were selected for further analysis. Within the top quartile of genes coexpressed with *PREX1*, those coding for signaling proteins, including receptors, kinases, phosphatases, GTPases, RhoGEFs, and RhoGAPs, among others, were identified by merging the data with a list of all signaling proteins obtained from the SMART platform (http://smart.embl-heidelberg.de/). It includes all human proteins containing phylogenetically conserved sequences predictive of structural domains with a role in intracellular signaling networks (40). The list was complemented with all the human G protein coupled receptors included in the UniProt platform (https://www.uniprot.org/; keyword/gpcr activity/human/reviewed).

#### Phosphosubstrate Datasets Analysis

The Phosphosite platform (https://www.phosphosite.org/homeAction), containing high throughput phosphoproteomic datasets, was analyzed to identify relevant substrates of the kinases found within the list of signaling proteins highly coexpressed with *PREX1*. Specifically, the kinase-substrate dataset, downloaded from the Phosphosite platform, containing substrates of 28 out of the 32 kinases that correlated with *PREX1* in the high expression list, was merged with the list of genes coexpressed with P-Rex1 in the high expression list.

#### Obtention and Validation of the PREX1 Signaling Hub Signature

The *PREX1* signaling hub signature, composed by *PREX1* and 30 signaling partners, was identified in the TCGA-LGG dataset by selecting signaling proteins highly coexpressed with *PREX1*, preferentially in the group of patients highly expressing this RacGEF. Only those signaling genes whose high expression had statistically significant correlation with shorter patient survival were included. The *PREX1* signaling hub signature was analyzed as a risk transcriptional signature for survival outcome by univariate Cox Proportional Hazards model in independent datasets of low grade glioma and other types of cancer. Initially, TCGA-LGG dataset, from which the signature was obtained, was analyzed using the Custom option of the KM plotter platform (https://kmplot.com/analysis/index.php?p=service&cancer=custom_plot) (41). Expression levels of each gene of the *PREX1* signature in each patient of the LGG dataset and their survival status were downloaded from the Oncolnc platform (http://www.oncolnc.org/; mRNA Expression, RSEM (Batch normalized from Illumina HiSeq_RNASeqV2), Excel file). To obtain the expression data of the *PREX1* hub signature in other TCGA datasets, each dataset was independently selected, and analyzed with the list of 30 genes pasted into the space for user-defined list in cBioportal. Data were downloaded as tab-delimited format and transferred to an Excel file, which was merged (using the patient’s ID) with a file containing the individual survival status, a single text file was imported into the KM plotter platform. The prognostic value of the *PREX1* signaling hub signature was validated with independent LGG datasets from the Chinese Glioma Genome Atlas (CGGA, http://www.cgga.org.cn/), from which we selected two different datasets (mRNAseq_693 batch 1 and mRNAseq_325 batch 2). The analysis in other TCGA studies, corresponding to different cancer types, included LUAD, KIRC and LAML, that initially had a correlation between *PREX1* expression and survival, and others without that previous correlation. The prediction was that the *PREX1* signaling hub signature would have a statistical correlation between expression and patient survival higher than *PREX1* by itself. Graphs were prepared in GraphPad Prism 6.01 and heatmaps were prepared in Clustvis (https://biit.cs.ut.ee/clustvis/ (42),).

#### Immune and Stromal Infiltration in LGG

The TCGA LGG dataset, divided in two groups by low and high *PREX1* expression, was analyzed with the ESTIMATE algorithm for immune, stromal and combined scores (43). The ESTIMATE values for LGG were extracted from the Merged Cohort of LGG and GBM (TCGA, Cell 2016), by selecting the information exclusive for LGG patients. Data were downloaded as three tab-delimited format files (https://www.cbioportal.org/study/summary?id=lgggbm_tcga_pub), inserted into Excel files, and merged with *PREX1* expression data using the patient’s ID. By diving the groups in low and high *PREX1* expression, we ended up with the immune, stromal and combined scores per group. Immune and stromal scores were statistically compared between groups.

#### Immune Gene Set Enrichment

The *PREX1* signaling hub was analyzed in the TIMER2.0 platform (http://timer.comp-genomics.org/) to assess whether this set of genes was indicative of immune cell infiltration in LGG. TIMER2.0 includes algorithms to address the fraction of different immune cell populations in the tumor microenvironment (44). It calculates survival outcomes with independent Cox regression models with TIMER (45), xCELL (46), and EPIC (43), among other algorithms. In addition, to find out which cell type signatures were compatible with the *PREX1* signaling hub, this set of genes was analyzed in the METASCAPE platform (47), with emphasis on the *cell type signatures* section (https://metascape.org/gp/index.html#/main/step1).

#### Cell Markers

To further address the contribution of cells of the tumor microenvironment to the repertoire of signaling transcripts that correlated with high *PREX1* expression and shorter patient survival, their Spearman’s correlation coefficients with established cell markers was analyzed. Markers included *GFAP* for astrocytes, *MBP* for oligodendrocytes, *TMEM119* for microglia, *ALDH1A1* for cancer stem cells, *PECAM1* for endothelial cells, *PTPRC* for leukocytes, *ITGAM* for macrophages, *CSPG4* for pericytes and *EPCAM* for epithelial cells (http://biocc.hrbmu.edu.cn/CellMarker/). Heatmap was prepared in Clustvis (https://biit.cs.ut.ee/clustvis/ (42),).

#### Statistical Analysis

Multiple comparisons were analyzed by ordinary one-way ANOVA followed by Tukey. Groups of two were analyzed by unpaired t tests with Welch’s correction. *PREX1* signaling hub validation was analyzed by univariate Cox Proportional Hazards model in KM plotter/Custom (http://www.kmplot.com/analysis/index.php?p=service). ANOVA and t tests were done with the GraphPad Prism 6.01 software, which was also used to prepare the graphs.

## Results

### High *PREX1* Expression Correlates With Shorter Survival of Brain Lower Grade Glioma Patients

**Figure 1:**
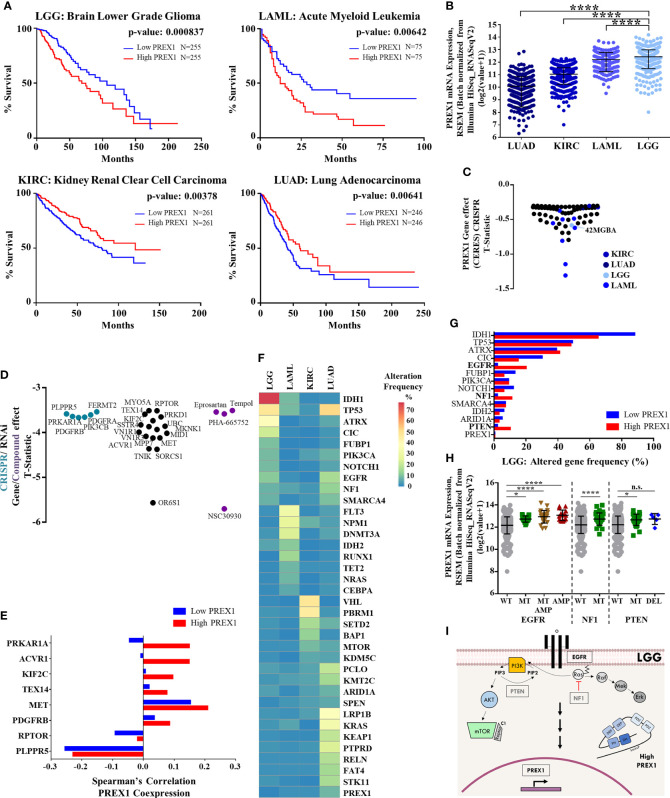
High *PREX1* expression correlates with shorter LGG patient survival. **(A)** High *PREX1* expression assessed in TCGA datasets correlates with shorter overall survival of LGG, LAML, KIRC and LUAD patients. Tumors with highest and lowest *PREX1* expression (50:50 percentiles) were compared. mRNA expression values [RSEM (Batch normalized from Illumina HiSeq_RNASeqV2)] were used for the analysis. Statistical p values were calculated using the Log-rank test. **(B)** Comparative analysis of *PREX1* mRNA expression (RNAseq) among LGG, LAML, KIRC and LUAD tumors, with data from TCGA studies obtained from the cBioPortal platform. Error bars represent mean ± SD. ****p < 0.0001, one way ANOVA followed Tukey. **(C)** *PREX1* dependent cell lines analyzed in the DepMap portal representing LGG, LAML, KIRC and LUAD cancer types. Graph shows T-statistic values. Different tones of blue were assigned to cell lines corresponding to each cancer type. **(D)** Astrocytoma dependencies revealed by CrisprCas9 (blue green), RNAi (black), and small molecules (purple) targeting the signaling repertoire. Effects are displayed as T-statistic values from the DepMap platform. **(E)** Coexpression of *PREX1* and selected essential signaling proteins, identified in cell lines within the astrocytoma context [shown in **(D)**], was compared between low and high *PREX1*-expressing LGG tumors of the TCGA transcriptomic dataset. **(F)** Comparative mutational signature of the top ten altered genes in LGG, LAML, KIRC and LUAD tumors. Scale shows percent of alteration. **(G)** Top ten frequencies of altered genes in LGG tumors with low and high *PREX1* expression. Genes with higher alteration frequency in tumors with high *PREX1* expression are highlighted in bold fonts. **(H)** *PREX1* expression in LGG tumors with wild type (WT), mutated (MT) and/or amplified (AMP) *EGFR, NF1* and *PTEN* genes. *EGFR* *p < 0.05, ****p < 0.0001, one way ANOVA followed Tukey. *NF1*. ****p < 0.0001, t test. *PTEN*. *p < 0.05, one way ANOVA followed Tukey. n.s., non-significant. **(I)** Model showing canonical EGFR, NF1 and PTEN signaling pathways and their putative impact on *PREX1* expression.

Given the metastatic role of P-Rex1 demonstrated in preclinical cancer models (6–9), we analyzed 21 TCGA transcriptomic datasets to identify those cancer types in which patient survival correlated with *PREX1* expression. Only lower grade glioma (LGG) and acute myeloid leukemia (LAML) showed reduced survival among the group of cancer patients with high *PREX1* expression, while kidney renal clear cell carcinoma (KIRC) and lung adenocarcinoma (LUAD) showed the opposite relationship, a better survival for the high *PREX1* expression groups (**Figure 1A**). *PREX1* mRNA expression values, obtained from the cBioPortal for Cancer Genomics, were higher in LGG and LAML patients compared to KIRC and LUAD (**Figure 1B**). To know whether P-Rex1 expression is essential in cancer cell lines prototypical of neoplasias in which this RhoGEF correlated with patient survival, we analyzed its essentiality profile. As shown in **Figure 1C**, LGG and LAML cell lines, but not KIRC or LUAD cell lines, had T-Statistic values below -0.5, indicating that P-Rex1 contributes to proliferation and survival of glial and acute myeloid leukemia cell lines (**Figure 1C**). Since LGG had the highest correlation between high *PREX1* expression and reduced patient survival, we focused our essentiality analysis on the astrocytoma context, which includes the 42MGBA cell line, to reveal in detail its essentiality profile of genes coding for signaling proteins, aiming to identify potential P-Rex1 partners in LGG oncogenic settings. Data available in the DEPMAP platform included effects of RNAi, Crispr-Cas9 and small molecules on the growth and survival of multiple cell lines. We identified 26 essential genes, coding for signaling proteins, with T-Statistic values below -3 (**Figure 1D**). These included tyrosine kinase receptors (*MET*, *PDGFRA/B*), cytosolic lipid kinases and protein kinases (*PIK3CB*, *PRKD1*) and protein kinase regulatory subunits (*PRKARIA*, *RPTOR*).

The hypothetical role of P-Rex1 as a functional signaling platform, putatively assembling a network with receptors, kinases and phosphatases, among other signaling partners, was defined as the network of coessential signaling proteins identified in the cancer cells in which P-Rex1 was essential, and the set of signaling proteins highly coexpressed with *PREX1* in patients in which their expression correlated with shorter survival. The 26 essential signaling genes, identified in the P-Rex1-dependent astrocytoma cell context, were further examined in terms of their co-expression with *PREX1* in LGG patients. Transcriptomic data of LGG patients, grouped based on *PREX1* expression (High *PREX1* and Low *PREX1* in **Figure 1A**), were mined to identify the potential correlation with *PREX1* expression of these astrocytoma essential signaling genes, according to Spearman’s correlation coefficient. We predicted that those highly correlated (in the high *PREX1* expression group), would be more linkable to hypothetical malignant signaling cascades integrated by *PREX1*. A previously identified P-Rex1-interacting partner (48, 49), *PRKAR1A*, the gene coding for the regulatory subunit of protein kinase A, showed the highest difference of Spearman’s among the low and high *PREX1* expression sets, followed by other five genes (**Figure 1E**), including *MET* and *PDGFRB*, coding for growth factor receptors known to activate P-Rex1-dependent cascades (50, 51).

### *EGFR*, *NF1* and *PTEN* Alteration Correlates With High *PREX1* Expression in Brain Lower Grade Glioma

To look for genetic alterations that might be linked to increased *PREX1* expression, we first compared the frequency of the top ten mutant genes in LGG, LAML, KIRC, and LUAD; those cancer types that had statistical significance of *PREX1* expression and patient survival. The mutational landscape among these cancer types was different and only few altered genes were common, particularly between LGG and LUAD, which coincided in *TP53*, *EGFR* and *NF1* mutation frequency (**Figure 1F**). *PREX1* itself was rarely found mutated, indicating that its high expression in patients with reduced survival was likely linked to their mutational background which somehow promoted *PREX1* expression. We then compared the mutational frequency of LGG patients with low and high *PREX1* expression. We found that alterations on *EGFR* (2% to 20%), *NF1* (2% to 11%) and *PTEN* (2% to 10%) genes were more frequent in the high *PREX1* expression group (**Figure 1G**). The increased mutational frequency of *EGFR* (a tyrosine kinase receptor), *NF1* (a RasGAP) and *PTEN* (a phosphatidylinositol (3,4,5)-trisphosphate phosphatase) in tumor samples of patients with high *PREX1* expression suggested that these oncogenic drivers, known to contribute to the classification of a subpopulation of LGG patients (52, 53), might regulate *PREX1* expression. To further sustain this possibility, we selected the group of patients that had at least one of these three genes altered (**Figure 1H**). Tumors with mutated or amplified *EGFR, NF1* or *PTEN* exhibited higher expression of *PREX1* (except in the cases with deleted *PTEN*), (**Figure 1H**), which is consistent with the possibility that canonical growth-factor regulated signaling cascades promote *PREX1* expression (**Figure 1I**).

### Signaling Companions of Highly Expressed *PREX1* in Brain Lower Grade Glioma

**Figure 2:**
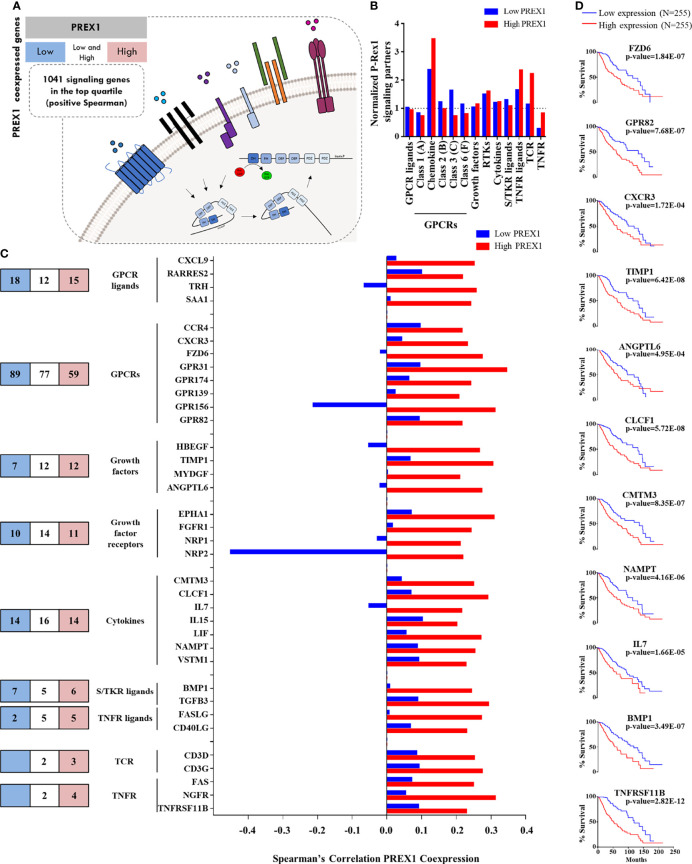
Agonists and receptors highly coexpressed with *PREX1* in LGG. **(A)** Model representing the strategy to identify *PREX1* signaling partners in LGG tumors highly expressing this RacGEF. The LGG TCGA transcriptomic dataset was divided in low and high *PREX1* expression groups. Potential P-Rex1 signaling partners were identified in the top quartile of coexpressed genes by merging the data with a list of all human signaling proteins tagged by their functional characteristics. The list included agonists and receptors (this figure), kinases and phosphatases (**Figure 3**) and a diversity of other signaling proteins (**Figure 4**). **(B)** Normalized coexpression of *PREX1* with agonists and receptors within the fourth quartile of high and low expression groups. Number 1.0 indicates that the number of coexpressed signaling proteins is the same as one quarter of existing transcripts for the indicated group. **(C)** Number of agonists and receptors in the top quartile of genes coexpressed with *PREX1* identified in the low (blue), high (red) or both (white) coexpression groups. The graph at the right compares the coexpression with *PREX1* of agonists and receptors in the high and low expression subsets. Only those with higher Spearman’s correlation value with *PREX1* in the high expression group are included. **(D)** Overall survival curves based on the expression of agonists and receptors highly coexpressed with *PREX1* (50:50 percentiles). Statistical significance was analyzed by the Log-rank test.

**Figure 3:**
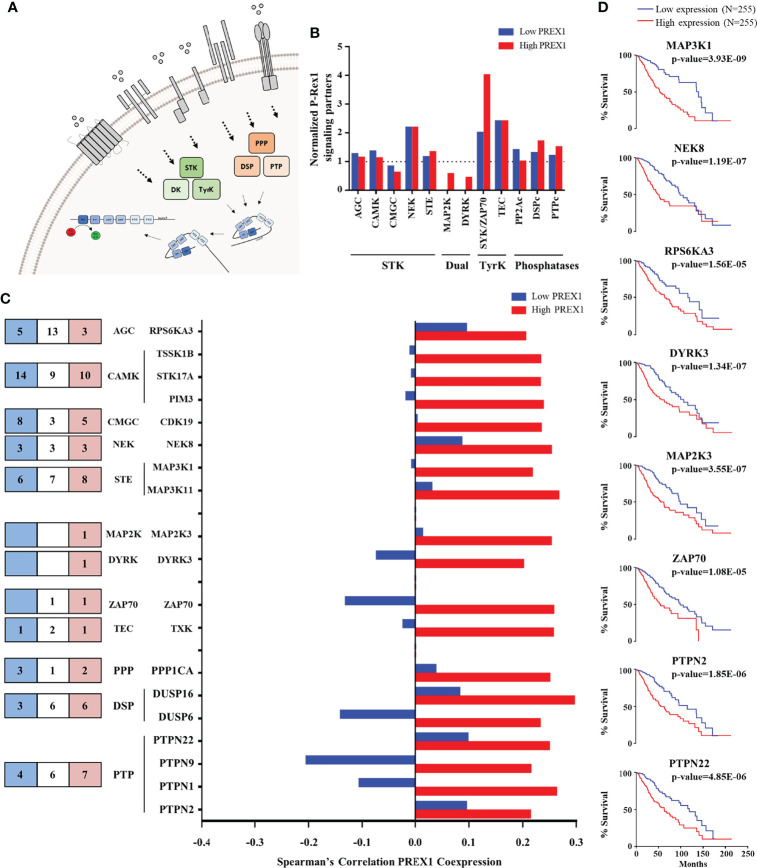
Kinases and phosphatases highly coexpressed with *PREX1* in LGG. **(A)** Model depicting serine/threonine kinases (STK), tyrosine kinases (TyrK) and kinases with dual specificity (DK) and phosphatases (PPP, PTP and DSP) potentially linked to P-Rex1 signaling. **(B)** Normalized coexpression with *PREX1* and protein kinases and phosphatases within the top quartile of *PREX1* coexpression list. A value of 1.0 indicates that the number of coexpressed genes in low and high *PREX1* coexpression lists (blue and red), within the top quartile, equals one-fourth of the existing genes within the indicated group of signaling proteins. **(C)** Number of the indicated groups of kinases and phosphatases highly coexpressed with *PREX1* only in the low (blue), high (red) or both (white) *PREX1* coexpression lists (left). Those preferentially coexpressed with *PREX1* within the high expression group (red bars) are indicated in the graph (right). **(D)** Overall survival curves based on the expression of protein kinases and phosphatases highly coexpressed with *PREX1* (50:50 percentiles). Statistical significance was assessed by the Log-rank test.

**Figure 4:**
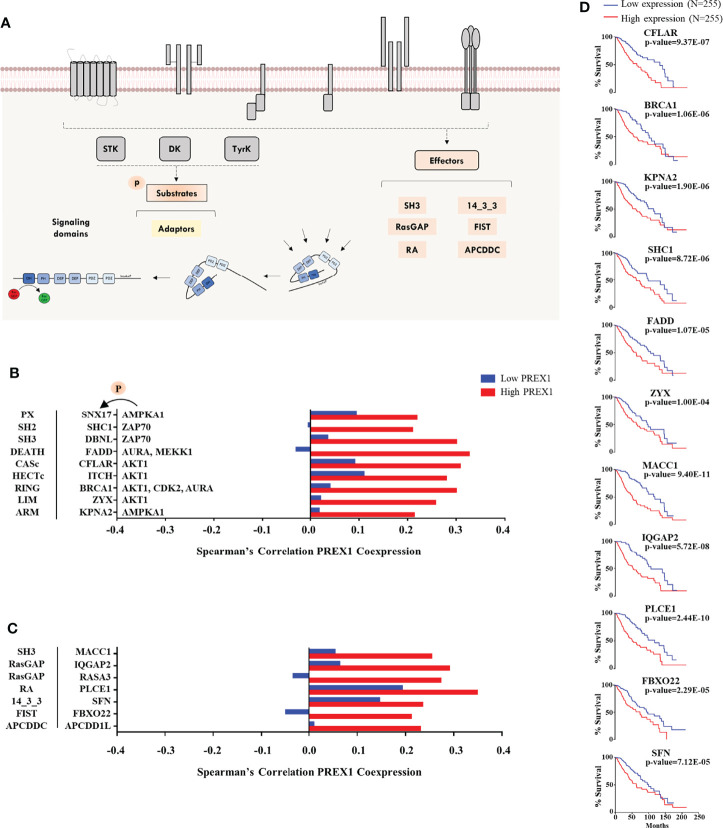
Additional signaling proteins within the repertoire of signaling transcripts highly coexpressed with *PREX1* in LGG. **(A)** Model showing phosphoproteins and effectors potentially linked to P-Rex1 signaling. **(B)** Transcripts coding for phosphoproteins (identified as such in the Phosphosite platform) highly coexpressed with *PREX1* in the top quartile of the high *PREX1* expression group. Their most relevant signaling domains are indicated at the left and their upstream kinases at the right. **(C)** Diverse signaling proteins and effectors highly coexpressed with *PREX1* in the high *PREX1* expression set. Their main signaling domains are indicated at the right. Bars represent Spearman’s correlation coefficients in the low and high *PREX1* expression lists (blue and red). **(D)** Overall survival of LGG patients distributed according to the expression of the indicated transcripts, highly coexpressed with *PREX1*, (50:50 percentiles). Statistical significance was analyzed by the Log-rank test.

We hypothesized that P-Rex1 constitutes a signaling hub that integrates receptors, transducers and effectors as functional modules relevant for the outcome of cancer patients. Thus, to identify the components of that putative signaling hub, we analyzed the TCGA LGG dataset, divided in two groups, organized as low and high *PREX1* expression, corresponding to those with longer and shorter patient survival, respectively, as shown in **Figure 1A**. We obtained two independent lists of genes, based on their coexpression with *PREX1*, with Spearman’s correlation coefficients ranging from negative to positive values, indicating various levels of coexpression with this RacGEF. Aiming to characterize the signaling transcriptome that accompanies highly expressed *PREX1*, we mined these coexpression gene lists. We compared the high *PREX1* expression list (20,096 genes) with the low expression list (20,095 genes) and divided each group in 4 quartiles (around 5,000 genes per list). We focused on positively correlated genes, coding for signaling proteins, that might be considered P-Rex1 signaling partners (the ranges of Spearman’s correlation coefficients in the top quartiles of the low and high *PREX1* expression groups were: 0.11-0.53, and 0.11-0.51, respectively). To identify which genes code for signaling proteins, we merged the list of coexpressed genes with a list of all human proteins that contain phylogenetically conserved domains consistent with their participation in intracellular signaling networks. These lists were obtained from the SMART and UniProt internet platforms (as described in methods). We identified 1,041 genes coding for signal transduction proteins coexpressed with *PREX1* in top quartile of the high expression group (**Figure 2A**). Since the high *PREX1* expression group correlated with shorter patient survival, we focused on signaling genes preferentially found in this group, in contrast with the low expression group. The identified proteins included signaling categories such as G protein coupled receptors, tyrosine kinase receptors, cytosolic serine/threonine and tyrosine kinases and phosphatases, and proteins with diverse protein-protein interaction domains, among others, as shown in **Figures 2**–**4**. We considered that a particular set of signaling proteins was preferentially coexpressed with *PREX1* when more than one fourth of its members was found in the top quartile of *PREX1* coexpressed genes (**Figures 2B**, **3B**).

Since a prototypical signaling cascade starts with the interaction between extracellular agonists and their receptors, followed by the activation of intracellular transducers and effectors, in **Figures 2**–**4** we present the potential P-Rex1 signaling partners in the order in which they would participate in a signaling cascade. Agonists and receptors are presented in **Figure 2**, kinases and phosphatases in **Figure 3**, phosphosubstrates of the kinases coexpressed with *PREX1* and proteins with additional signaling domains in **Figure 4**. In all the cases, we included genes coding for signaling proteins that had positive Spearman correlation coefficients of coexpression with *PREX1*, preferentially in the high expression group. We considered part of the *PREX1* signaling hub only those signaling partners that had a statistically significant correlation with shorter patient survival.

### Chemotactic Agonists, Growth Factors and Receptors in the Group of LGG Patients With Highly Expressed *PREX1*

Given that P-Rex1 is activated in response to growth factors and extracellular chemotactic cues (**Figure 2A**), we aimed to identify chemotactic agonists, growth factors and receptors coexpressed with this RacGEF, particularly looking for those with higher correlation within the group of high *PREX1* expression that might be indicative of shorter survival of LGG patients. Chemotactic GPCRs were preferentially enriched in the high *PREX1* expression group (**Figure 2B**). A substantial number of transcripts coding for extracellular factors, chemokines, cytokines, and receptors were found in the highest quartile of genes coexpressed with *PREX1*. The blue and red areas in **Figure 2C**, left panel, indicate the numbers of transcripts of selected groups of signaling proteins exclusively present in the highest quartile of the low and high *PREX1* expression groups, respectively, whereas those in the white areas were present in the top quartile of both groups, G protein coupled receptors (GPCRs) and growth factor receptors were the most abundant (**Figure 2C**, left panel). The graph in **Figure 2C** (right) only shows agonists and receptors with contrasting correlations, in the high and low expression groups, with *PREX1* (and at least 0.2 Spearman coefficient correlation with *PREX1* in the high expression group). Among the transcripts coding for peptidic agonists, we identified GPCR ligands, such as the *CXCL9* chemokine, and growth factors known as agonists of tyrosine kinase receptors (*HBEGF, ANGPTL6*, **Figure 2C**), cytokines (*IL7, IL15*, **Figure 2C**), and agonists of serine/threonine kinase receptors (*BMP1*, *TGFB3*, **Figure 2C**). Of the GPCRs and their agonists that showed clear contrast in their Spearman correlation values comparing the high and low *PREX1* expression groups, we found a known chemokine-GPCR pair, *CXCL9-CXCR3*, already described as relevant in metastatic cancer progression (54). The high *PREX1* expression group included 4 growth factor receptors that showed higher Spearman’s coefficient in the high versus the low *PREX1* expression groups; this set included ephrin (*EPHA1*), *FGFR1*, and VEGF receptors (*NRP1/2*) (**Figure 2C**). In prostate cancer, *NRP2*, mediates bevacizumab resistance *via* a VEGF/NRP2/PREX1/RAC1 pathway (55). Other receptor classes with higher correlation with *PREX1* within this group were TCRs and TNFRs (**Figure 2C**). When highly expressed, seven extracellular peptides (*TIMP1, ANGPTL6, CLCF1, CMTM3, NAMPT, IL7* and *BMP1*), and four receptors (*FZD6*, *GPR82*, *CXCR3*, and *TNFRSF11B*), correlated with shorter survival of LGG patients at the same or more significant degree as *PREX1* (Logrank p-value), (**Figure 2D**).

### Kinases and Phosphatases Within the Top Quartile of *PREX1* Coexpressed Genes in LGG Patients With Shorter Survival

Regarding signaling effectors potentially linked to P-Rex1, kinases and phosphatases are particularly interesting because this RacGEF is known to be regulated by phosphorylation (**Figure 3A**) and direct interaction with mTOR and PKA kinases (48, 49, 56). The top quartile of *PREX1* coexpressed genes included 5 families of serine/threonine kinases, 2 of dual kinases and 2 of cytosolic tyrosine kinases (**Figures 3B, C**, left panel). The cytosolic ZAP70 kinase was particularly enriched in the high *PREX1* expression group (**Figure 3B**). It also included members of the three known groups of phosphatases (PPPs, DSPs and PTPs; **Figures 3B, 3C**, left panel). Kinases and phosphatases with at least a Spearman correlation value of 0.2 with *PREX1* in the high expression group are included in the graph shown in **Figure 3C**. Survival curves based on the expression of kinases (*MAP3K1, NEK8, RPS6KA3, DYRK3, MAP2K3*, and *ZAP70*), and phosphatases (*PTPN2* and *PTPN22*), which correlated with shorter LGG patient survival when highly expressed, are shown in **Figure 3D**.

### Potential Substrates of P-Rex1-Linked Kinases and Prototypical Signaling Hardware Coexpressed With P-Rex1

Phosphoproteomic datasets provide the identity of known substrates of specific kinases. Given the identification of highly coexpressed P-Rex1-linked kinases, we looked for the presence of their known substrates within the top quartile of *PREX1* coexpressed genes (**Figure 4A**, left) and signaling effectors (**Figure 4A**, right) characterized by the presence of relevant structural domains. This group was constituted by miscellaneous proteins, including Rho regulators, adaptor proteins and other signaling hardware identified by the presence of structural domains consistent with their participation in intracellular signaling processes, as described in the SMART platform (57). They are presented in **Figure 4B** (phosphosubstrates) and **Figure 4C** (other signaling hardware) organized by their main signaling domain indicated at the left and, in the case of known phosphosubstrates, the corresponding kinases are indicated at the right of their targets (**Figure 4B**). With this approach, we identified potential P-Rex1 signaling partners containing PX, SH2, and SH3 domains (**Figure 4B**), and effectors tagged by their structural signaling domains. Among the coexpressed genes in the high *PREX1* expression group, we found regulators of small Rho and Ras GTPases (*IQGAP2, RASA3*), a 14-3-3 adaptor protein (*SFN*), an ubiquitin ligase (*FBXO22*), and phospholipase-C epsilon (*PLCE1*) which, as P-Rex1, is a Gβγ effector (58). Eleven transcripts of signaling proteins presented in **Figures 4B** and **4C** exhibited correlation with shorter patient survival when highly expressed (**Figure 4D**).

### P-Rex1 Signaling Hub Validation in Independent LGG and TCGA Datasets

**Figure 5:**
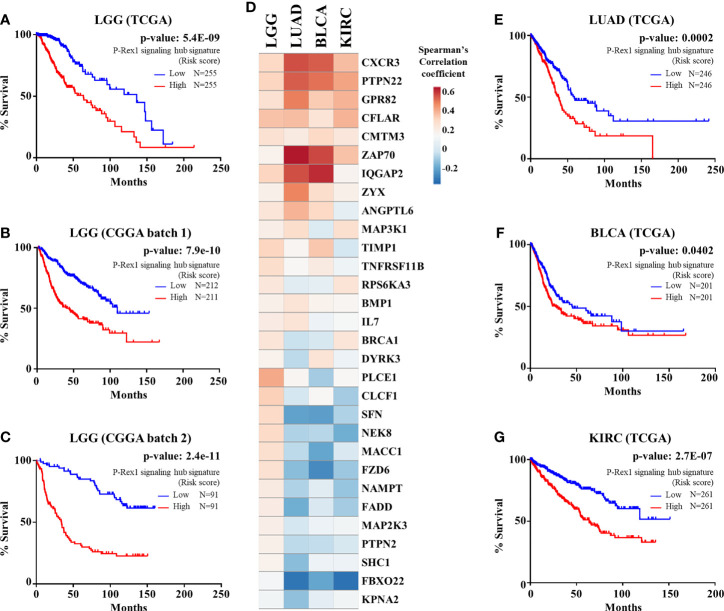
*PREX1* signaling hub validation in independent datasets. **(A)** Overall survival of LGG patients of the TCGA study based on the risk score defined by the *PREX1* signaling hub signature. **(B, C)** The predictive value of the *PREX1* signaling hub signature, discovered in the LGG dataset of the TCGA study, was validated with independent LGG datasets of the CGGA study: **(B)**, batch 1, and **(C)**, batch 2. **(D)** Comparative coexpression of *PREX1* and its signaling partners, that constitute the *PREX1* signaling hub signature, among LGG, LUAD, BLCA, and KIRC tumors of the TCGA studies (comparison among all 32 TCGA studies is shown in **Supplementary Figure 1**). **(E–G)** Overall survival curves of LUAD **(E)**, BLCA **(F)**, and KIRC **(G)** patients divided by their high and low risk scores defined by the *PREX1* signaling hub signature. Analysis of the overall survival of cancer patients, based on the risk score defined by the *PREX1* signaling hub signature, was done by the univariate Cox Proportional Hazards model.

We considered the 30 signaling partners of *PREX1*, that by themselves had a significant correlation with shorter patient survival, as part of a putative signaling hub signature with potential predictive value in LGG. To address this possibility, we first analyzed, by univariate Cox Proportional Hazards model, its combined risk score for survival outcome in the group of TCGA-LGG patients in which the *PREX1* signaling hub signature was discovered. As shown in **Figure 5A**, the *PREX1* signaling hub signature significantly correlated with increased risk of shorter survival in TCGA LGG patients. This finding was confirmed in independent LGG datasets and other types of cancer of the TCGA project. Analysis of the predictive value of the *PREX1* signaling hub signature in two independent LGG datasets from the Chinese Glioma Genome Atlas (CGGA) (59) demonstrated a significant high risk score that correlated with shorter survival (**Figures 5B, C**).

Next, we analyzed how the 30 genes discovered as components of the *PREX1* signaling hub signature correlated, in terms of expression, with this RacGEF in other TCGA studies. Since various genes of the signature positively correlated with *PREX1* in more than 20 types of cancer (**Supplementary Figure 1**), we addressed whether, as an integrated signature, it revealed increased risk in these independent datasets. The heatmap shown in **Figure 5D** displays the comparative coexpression of *PREX1* and its 30 signaling partners in LGG, LUAD, BLCA (bladder urothelial carcinoma) and KIRC, TCGA datasets. As in the case of LGG (**Figures 5A–C**), the *PREX1* signaling hub signature revealed a significant increased risk score in LUAD, BLCA and KIRC (**Figures 5E–G**). Notably, *PREX1* by itself correlated with longer survival in LUAD and KIRC studies (**Figure 1A**), whereas the *PREX1* signaling hub signature had the opposite predictive value, consistent with the result in LGG, and also revealed an increased risk of shorter survival in BLCA, which did not have a significant correlation with *PREX1* expression by itself. These finding revealed the power of the *PREX1* signaling hub signature, beyond the potential of analyzing the expression of only this RacGEF. We also found that the *PREX1* signaling hub signature indicated higher risk score for shorter survival in uveal melanoma and uterine carcinosarcoma (TCGA studies), which were not initially addressed as they were not included in the OncoLnc platform that we used for the initial screening.

### P-Rex1 Signaling Hub in LGG Patients With Reduced Survival Correlates With Immune and Endothelial Markers

**Figure 6:**
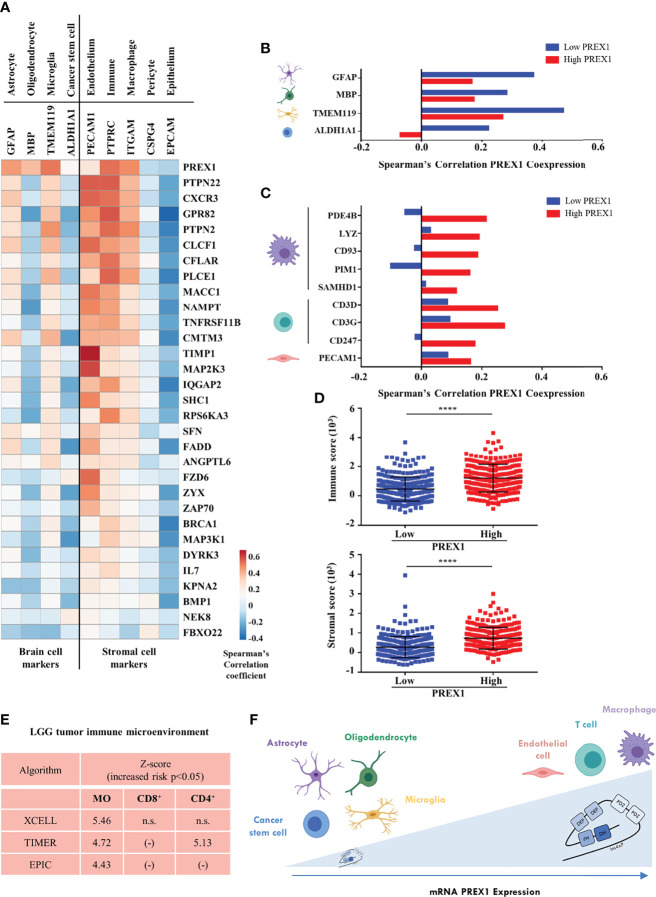
P-Rex1 signaling partners correlate with markers of the cell stroma in LGG tumors. **(A)** Coexpression of genes of the *PREX1* signaling hub signature with markers of different cells within the LGG tumor microenvironment. Blue and red colors indicate the Spearman’s correlation coefficient scale from negative to positive values. **(B)** Coexpression of *PREX1* with cell markers of astrocyte, oligodendrocyte, microglial and cancer stem cells in the low and high *PREX1* expression subsets. **(C)** Coexpression of *PREX1* with markers of macrophage, T cell and endothelial cells in the low and high *PREX1* expression subsets. **(D)** Immune and stromal scores, predicted by the ESTIMATE algorithm, in tumors of LGG patients divided by low and high *PREX1* expression, ****p < 0.0001, t test. **(E)** Overall analysis of LGG tumor immune microenvironment as estimated by the indicated algorithms. Significant values indicative of the presence of macrophages (MO), and T lymphocytes (CD8^+^ and CD4^+^) are shown. Z-scores indicate an increased risk of shorter survival (p < 0.05), n.s., not significant. **(F)** Model depicting the LGG tumor microenvironment showing the presence of different cell types correlated with *PREX1* expression (horizontal arrow). Tumors with higher *PREX1* expression exhibit increased immune infiltration and microvasculature.

We identified 30 putative signaling partners of highly expressed P-Rex1 that, as in the case of this RacGEF, were statistically linked to shorter LGG patient survival. Next, we analyzed whether expression of these genes was indicative of the complexity of the tumor microenvironment by their correlation with markers of cells within the tumor and stroma, in the LGG context. These markers represent different cell types, including immune, endothelial and cancer cells (**Figure 6A**). The first row indicates that *PREX1* was expressed in most cells of the tumor microenvironment. Most P-Rex1 signaling companions correlated positively with stromal cell markers, including leukocytes, myeloid, and endothelial cells (**Figure 6A**, right side). A fraction of *PREX1* signaling partners exhibited good correlation with astrocyte and microglia markers and negative expression in cancer stem cells and oligodendrocytes (**Figure 6A**, left side). Within the group of transcripts coexpressed with *PREX1*, we looked for those previously reported as cell markers (60). Consistent with an increased heterogeneity of stromal cells in tumors highly expressing *PREX1*, a contrasting correlation with brain cell markers and stromal cell markers was evident between the low and high *PREX1* expression groups (**Figures 6B, C**). The low *PREX1* expression group exhibited higher coexpression with brain cell markers (**Figure 6B**); whereas the high *PREX1* expression group had better correlation of this RacGEF with immune and endothelial cell markers (**Figure 6C**). We also analyzed the immune and stromal score of high and low *PREX1* expression groups. With the ESTIMATE algorithm for immune, stromal and combined scores (43), we found that the high *PREX1* expression group had higher immune and stromal scores (**Figure 6D**). To investigate whether the P-Rex1 signaling hub signature is indicative of immune infiltration and correlated with patient outcome, we analyzed the components of the signature as an integrated unit and tested its consistency with different cell signatures in the METASCAPE platform (47). As shown in **Supplementary Figure 2**, most of the identified cells were of myeloid origin. This result was consistent with the analysis of immune infiltration estimated in the TIMER2.0 platform (44), which using independent algorithms applied to the whole LGG dataset predicted a significant infiltration of macrophages (MO) and T cells (CD8^+^) by the XCELL, TIMER and EPIC algorithms (43, 44, 46), while CD4^+^ T cells were estimated by the TIMER algorithm (**Figure 6E**). Based on *PREX1* expression and patient survival, our approach guided us to identify a set of *PREX1* coexpressed transcripts coding for signaling proteins that marked stromal cell types, which we postulated as indicative of the existence of a P-Rex1 signaling hub in immune and endothelial cells in the group of patients with shorter survival (**Figure 6F**, upper right).

## Discussion

*PREX1* encodes a multidomain activator of the Rac GTPase known to promote cell migration and metastatic dissemination of cancer cells (5–9). Given its pathological implications in cancer progression and its complex architecture, we postulated P-Rex1 as a potential prognostic signaling hub relevant in various cancer settings. Aiming to recognize those cancer types in which *PREX1* expression correlates with shorter patient survival and identify its potential signaling partners, we analyzed transcriptomic datasets of 21 TCGA studies (https://www.cancer.gov/tcga). Lower Grade Glioma was the type of cancer in which half of the patients with higher *PREX1* expression exhibited the highest statistical correlation with shorter survival. Others in which a significant correlation was observed were acute myeloid leukemia in which, as in the case of LGG, elevated *PREX1* expression correlated with shorter survival, and kidney renal clear cell carcinoma and lung adenocarcinoma that had the opposite correlation. The contrast among these tumor contexts is consistent with the idea that highly expressed P-Rex1 might integrate differential, cancer-specific, signaling cascades. It might also be indicative of P-Rex1 expression in non-cancerous cells, reflecting cellular heterogeneity of tumors that might have an impact on tumor progression. Both possibilities are compatible with its potential as a cancer specific prognostic signaling hub. Since the best statistically significant correlation between highly expressed *PREX1* and shorter patient survival was observed in LGG patients, we carried out a detailed analysis of LGG transcriptomic datasets aiming to identify potential signaling partners of *PREX1* among the coexpressed transcripts that also had significant correlation with patient survival. In addition, we looked for cancer cell lines in which P-Rex1 is essential with the idea to identify potential signaling partners among other essential proteins. We also analyzed which oncogenes were related to elevated *PREX1* expression.

Since P-Rex1 signaling is controlled by chemotactic receptors, transducers, kinases, phosphatases and adaptor proteins, among others (1, 61), we screened the repertoire of *PREX1* coexpressed genes looking for transcripts coding for proteins with prototypical signaling domains. We postulated that, among them, we would identify some that, as in the case of *PREX1*, would be more expressed in the group of patients in which high *PREX1* expression coincided with shorter survival. Potential P-Rex1 signaling partners were selected as those prevalent in the high *PREX1* expression group that also correlated with shorter patient survival and had contrasting correlation coefficients with *PREX1* among the low and high *PREX1* coexpression sets. We identified a list of 30 signaling proteins (**Figure 6A**). It includes receptors that have been mechanistically linked to cancer progression such as the G protein coupled receptors *CXCR3* and *FZD6*, linked to tumor cell growth, invasion and migration (62–64), and PTPN22, a protein tyrosine phosphatase associated with inhibition of antioncogenic immune response (65). Since *PREX1* is expressed in different cell types of LGG microenvironment, it potentially establishes differential signaling cascades in stromal cells that might contribute to aggravate the malignant process. As indicative of an enriched immune tumor microenvironment, we found *CXCR3*, *ZAP70* and *PTPN22*; whereas others, such as *TIMP1* and *MAP2K3*, had higher correlation with endothelial markers indicative of a tumor-induced angiogenic process (66). Some agonists and potential P-Rex1 signaling partners, *CLCF1* (67), *RPS6KA3* (RSK2) (68), *PTPN2* (69), and *PTPN22* (65), are currently studied as targets of anti-oncogenic precision therapies. Their identification with P-Rex1 is consistent with the general idea that, together with this multidomain activator of the Rac GTPase, they are part of an oncogenic signaling hub with potential prognostic value. Their elevated expression in LGG patients with shorter survival also highlights further opportunities for studies looking to their actual druggability in this type of brain cancer.

Our analysis of essentiality datasets confirmed the importance of P-Rex1 in LGG cells and resulted in a list of essential signaling proteins. Some of them are known P-Rex1 signaling partners, including the PKA regulatory subunit that has been described as a direct activator of P-Rex1 (49). Others included receptor tyrosine kinases, such as Met, the HGF receptor, known to activate P-Rex1 (51). These findings might be indicative of early alterations in the HGF/cMet system, which is known to play an important role in advanced glioma (70). We identified higher *PREX1* expression in patients with mutated *EGFR, PTEN* and *NF1*, suggesting that these oncogenes are upstream regulators of *PREX1* transcription, which is consistent with previous genetic screens in diffuse glioma (52, 53). Among these cascades, *EGFR* is known to unleash the PI3K-AKT-mTOR pathway, which activates P-Rex1-dependent signaling (56, 71, 72).

LGG, considered immunologically quiescent (73, 74), is aggravated by immune infiltration (74). Among the LGG infiltrating immune cell types, we identified that macrophage markers preferentially correlated with *PREX1* in the group of patients with shorter survival (**Figure 6C**). This might represent infiltrating subpopulations of tumor associated macrophages (TAMs) (75), in contrast with resident microglia (76), whose marker had a better correlation with P-Rex1 in the longer survival group. These findings were consistent with the analysis of the whole LGG transcriptome by independent algorithms and supports the concept that elevated immune infiltration is indicative of bad prognosis. Our analysis indicates that this process might be monitored by the expression of P-Rex1 and its signaling partners.

In conclusion our analysis of transcriptomic datasets filtered to identify only those coding for signaling proteins coexpressed with *PREX1*, all of them statistically correlated with shorter patient survival, guided us to discover a transcriptional signature of signaling proteins with potential prognostic value that might be mechanistically connected to P-Rex1 function. Its prognostic value was confirmed in independent LGG datasets (**Figures 5A–C**) and was also indicative of increased risk of shorter survival in lung, bladder, and kidney cancers (**Figures 5E–G**). As an integrated signaling module, the *PREX1* signaling hub signature deserves further characterization in the context of LGG biology, as well as in other types of cancer, given that members of the signature consistently were found coexpressed with *PREX1*. Although we did not directly demonstrate a direct regulation of P-Rex1 by its potential signaling partners, knowing their identity and statistical correlation with patient survival puts the focus on them for future studies. Our ongoing investigations are leading towards this goal, which is further supported by our previous findings in which we had characterized P-Rex1 as and interactor of mTORC2, AKT and PKA kinases and an effector of growth factor receptors such as Met, and chemotactic GPCRs (48, 49, 51, 56), also evidenced by the current data mining strategies. Further characterization of P-Rex1-linked signaling proteins will provide valuable information to validate a signaling signature integrated by P-Rex1 with potential as biomarkers and targets of combined therapies, which are critical elements to design successful personalized therapies.

## Data Availability Statement

Publicly available datasets were analyzed in this study. This data can be found here: https://cbioportal.org/ http://www.oncolnc.org/ http://smart.embl-heidelberg.de/ https://www.uniprot.org/ https://depmap.org/portal/depmap/ https://www.phosphosite.org/homeAction http://biocc.hrbmu.edu.cn/CellMarker/.

## Author Contributions

YB-N, GR-C, and JV-P conceptualization. YB-N, and JV-P formal analysis. YB-N, GR-C, and JV-P data curation. YB-N writing–original draft. JV-P writing–review and editing. JV-P supervision. JV-P project administration. GR-C and JV-P funding acquisition. All authors contributed to the article and approved the submitted version.

## Funding

Grants were from Conacyt (Grant No. 319283 to JV-P) and FORDECYT-PRONACES (Grant No. 1794 to GR-C). YB-N is a graduate student supported by a fellowship from Conacyt.

## Conflict of Interest

The authors declare that the research was conducted in the absence of any commercial or financial relationships that could be construed as a potential conflict of interest.

## Publisher’s Note

All claims expressed in this article are solely those of the authors and do not necessarily represent those of their affiliated organizations, or those of the publisher, the editors and the reviewers. Any product that may be evaluated in this article, or claim that may be made by its manufacturer, is not guaranteed or endorsed by the publisher.

## Supplementary Material

Supplementary Figure 1 Comparative coexpression of *PREX1* with members of its signaling hub signature among 32 TCGA studies. Coexpression of *PREX1* with genes coding for members of the *PREX1* signaling hub signature was analyzed in the 32 TCGA studies in the cBioPortal platform. The figure shows the comparative coexpression lists displayed based on the Spearman’s correlation coefficients to present those with highest values at the top-left.

Supplementary Figure 2 The P-Rex1 signaling hub signature correlates with transcriptional signatures characteristic of myeloid cells. The *PREX1* signaling hub signature was analyzed in the METASCAPE platform to identify the cell types, defined by characteristic transcriptional signatures, that express a significant number of the same genes (77). Myeloid and endothelial cells were preferentially identified with the *PREX1* signaling hub signature.
