# Supplementary figures and images for: P-Rex1 Signaling Hub in Lower Grade Glioma Patients, Found by In Silico Data Mining, Correlates With Reduced Survival and Augmented Immune Tumor Microenvironment
Source: Front Oncol. 2022 Jul 7;12:922025. doi: 10.3389/fonc.2022.922025 (PMC9300953; doi:10.3389/fonc.2022.922025)

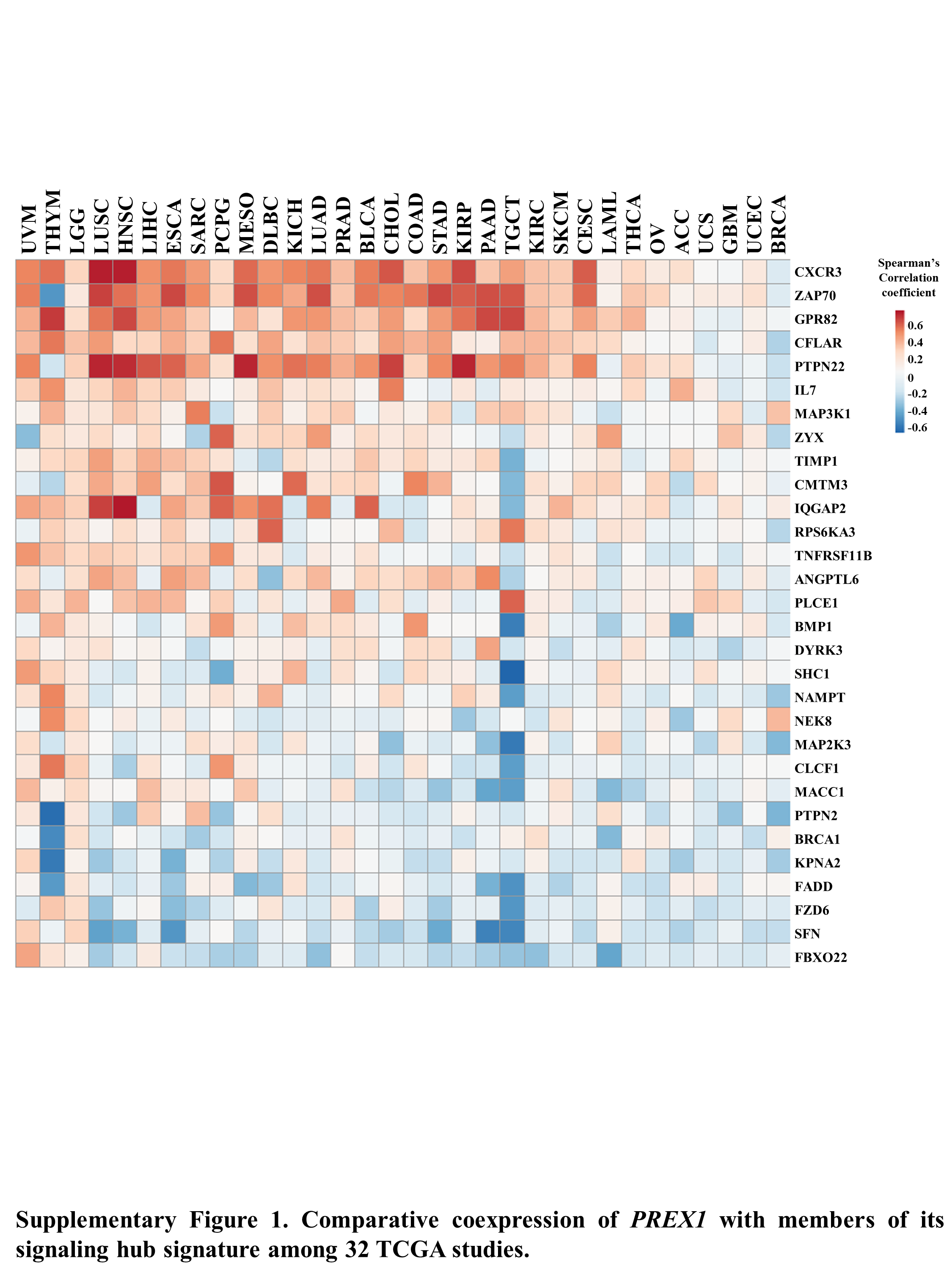

Supplement: Supplementary Figure 1 — Comparative coexpression of PREX1 with members of its signaling hub signature among 32 TCGA studies. Coexpression of PREX1 with genes coding for members of the PREX1 signaling hub signature was analyzed in the 32 TCGA studies in the cBioPortal platform. The figure shows the comparative coexpression lists displayed based on the Spearman’s correlation coefficients to present those with highest values at the top-left. [file Image_1.tif]

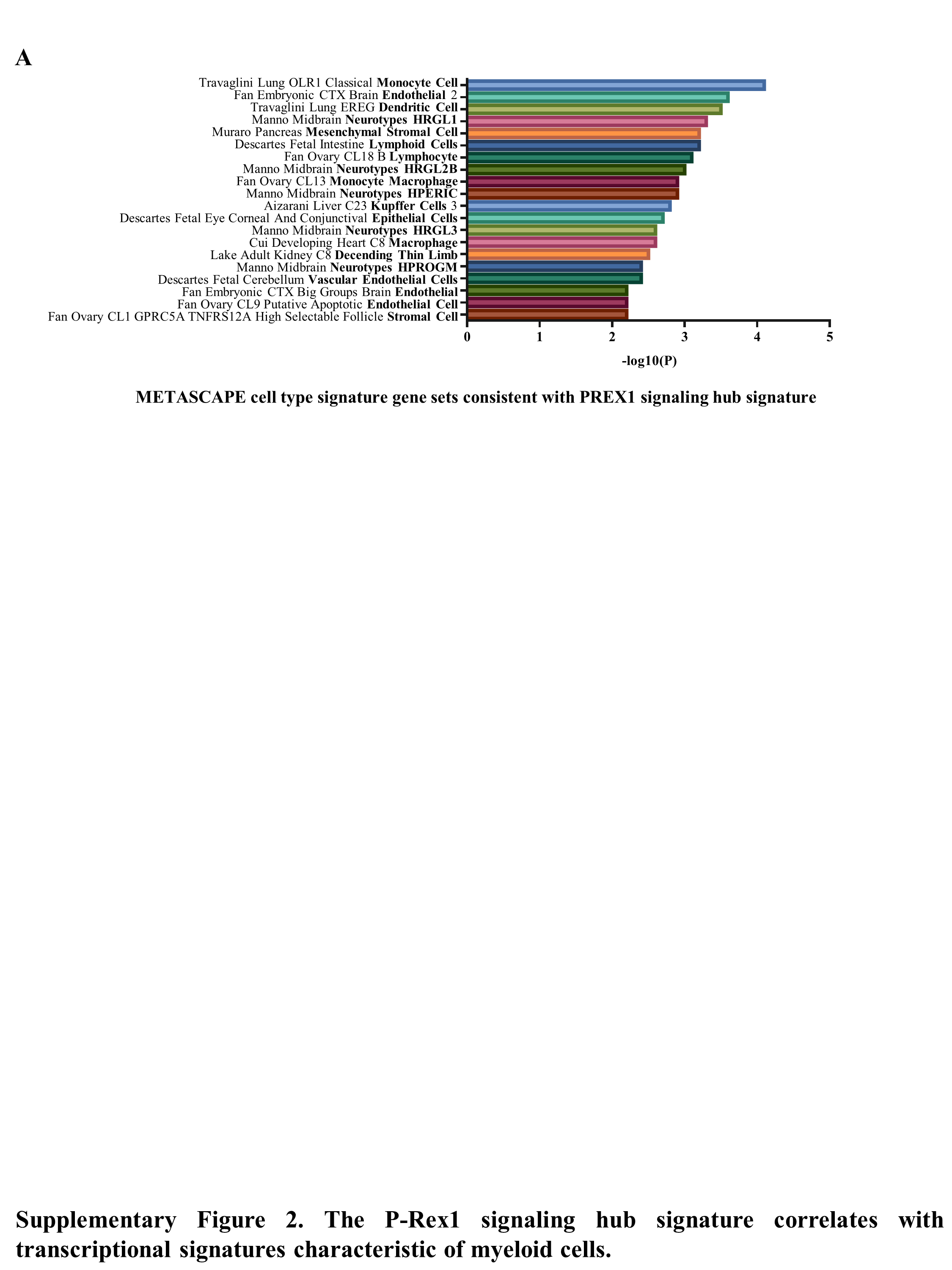

Supplement: Supplementary Figure 2 — The P-Rex1 signaling hub signature correlates with transcriptional signatures characteristic of myeloid cells. The PREX1 signaling hub signature was analyzed in the METASCAPE platform to identify the cell types, defined by characteristic transcriptional signatures, that express a significant number of the same genes (77). Myeloid and endothelial cells were preferentially identified with the PREX1 signaling hub signature. [file Image_2.tif]
